# Moderate and Deep Hypothermic Circulatory Arrest Have Comparable Effects on Severe Systemic Inflammatory Response Syndrome After Total Aortic Arch Replacement in Patients With Type A Aortic Dissection

**DOI:** 10.3389/fsurg.2021.758854

**Published:** 2021-12-06

**Authors:** Yingjie Du, Zhongrong Fang, Yanhua Sun, Congya Zhang, Guiyu Lei, Yimeng Chen, Lijing Yang, Xiying Yang, Jun Li, Guyan Wang

**Affiliations:** ^1^Department of Anesthesiology, Beijing Tongren Hospital, Capital Medical University, Beijing, China; ^2^Department of Anesthesiology, National Center of Cardiovascular Diseases, Chinese Academy of Medical Sciences and Peking Union Medical College, Fuwai Hospital, Beijing, China; ^3^Department of Anesthesiology, The Affiliated Drum Tower Hospital of Nanjing, University Medical School, Nanjing, China

**Keywords:** severe systemic inflammatory response syndrome, total aortic arch replacement (TAAR), moderate hypothermic circulatory arrest (MHCA), deep hypothermic circulatory arrest (DHCA), type A aortic dissection (TAAD)

## Abstract

**Background:** The objective of this study was to compare the incidence of severe systemic inflammatory response syndrome (sSIRS) after total aortic arch replacement between patients who underwent moderate hypothermic circulatory arrest (MHCA) and those who underwent deep hypothermic circulatory arrest (DHCA).

**Methods:** At Fuwai Hospital, 600 patients who underwent total aortic arch replacement with MHCA or DHCA from January 2013 to December 2016 were consecutively enrolled and divided into DHCA (14.1–20.0°C) and MHCA (20.1–28.0°C) groups. Preliminary statistical analysis revealed that some baseline indicators differed between the two groups; therefore, propensity score matching (PSM) was used to balance the covariates. Post-operative sSIRS as the primary outcome was compared between the groups both before and after PSM.

**Results:** A total of 275 (45.8%) patients underwent MHCA, and 325 (54.2%) patients underwent DHCA. After PSM analysis, a total of 191 matched pairs were obtained. The overall incidence of sSIRS was 27.3%. There was no significant difference in post-operative sSIRS between the MHCA group and the DHCA group in either the overall cohort or the PSM cohort (no-PSM: *P* = 0.188; PSM: *P* = 0.416); however, post-operative sSIRS was increased by ~4% in the DHCA group compared with the MHCA group in both the no-PSM and PSM cohorts (no-PSM: 29.5 vs. 24.7%; PSM: 29.3 vs. 25.1%). Both before and after PSM, the rates of gastrointestinal hemorrhage and pulmonary infection and post-operative length of stay were significantly increased in the DHCA group compared with the MHCA group (*P* < 0.05), and the remaining secondary outcomes were not significantly different between the groups.

**Conclusions:** MHCA and DHCA are associated with comparable incidences of sSIRS in patients following total aortic arch replacement for type A aortic dissection. However, the MHCA group had a shorter cardiopulmonary bypass time, a shorter post-operative length of stay and lower pulmonary infection and gastrointestinal hemorrhage rates than the DHCA group. We cautiously recommend the use of MHCA for most total arch replacements in patients with type A aortic dissection.

## Introduction

Post-operative systemic inflammatory response syndrome (SIRS) significantly increases morbidity, such as multiorgan failure, and even mortality (1, 2). Several factors, such as surgical trauma, blood contact with non-endothelial surfaces that leads to activation of the coagulation/complement cascade, ischemia-reperfusion, endotoxemia, hypothermia, blood transfusion, heparin and protamine, contribute to the development of SIRS after cardiac surgery with cardiopulmonary bypass (3–5). Severe systemic inflammatory response syndrome (sSIRS) (meeting all four criteria) rather than conventional SIRS (meeting two or more criteria) is more appropriate for predicting the relationships between the inflammatory response and its post-operative outcomes (1, 6).

More cooling, rewarming, and hypothermic circulatory arrest procedures are involved in total aortic arch replacement than in ordinary cardiopulmonary bypass; thus, inflammation may be more severe, and its incidence may be increased. Since 1975, deep hypothermic circulatory arrest (DHCA) has been widely accepted as the standard method for total aortic arch replacement (7). However, in recent years, with the clinical success of anterograde cerebral perfusion with moderate hypothermic circulatory arrest (MHCA), the need for DHCA for total arch replacement has been questioned (8).

To date, there are few studies comparing post-operative sSIRS in patients undergoing total arch replacement between DHCA and MHCA. The aim of this study was to compare the incidence of sSIRS following total arch replacement for type A aortic dissection between adult patients who underwent MHCA and those who underwent DHCA. Given that potential advantages of MHCA have been reported in several studies, we hypothesized that compared with DHCA, MHCA decreases the incidence of sSIRS in patients undergoing total arch replacement in type A aortic dissection.

## Materials and Methods

### Study Design and Ethical Statement

The study was approved by the Chinese Ethics Committee. Informed consent was waived due to the retrospective nature of the study. All procedures were carried out in accordance with the ethical standards of the 1964 Declaration of Helsinki and its subsequent amendments. This retrospective study was conducted in a series of adult patients with type A aortic dissection undergoing total aortic arch replacement with the use of the frozen elephant trunk technique at the National Cardiovascular Institute, Fuwai Hospital, Beijing.

### Study Population

**Figure 1:**
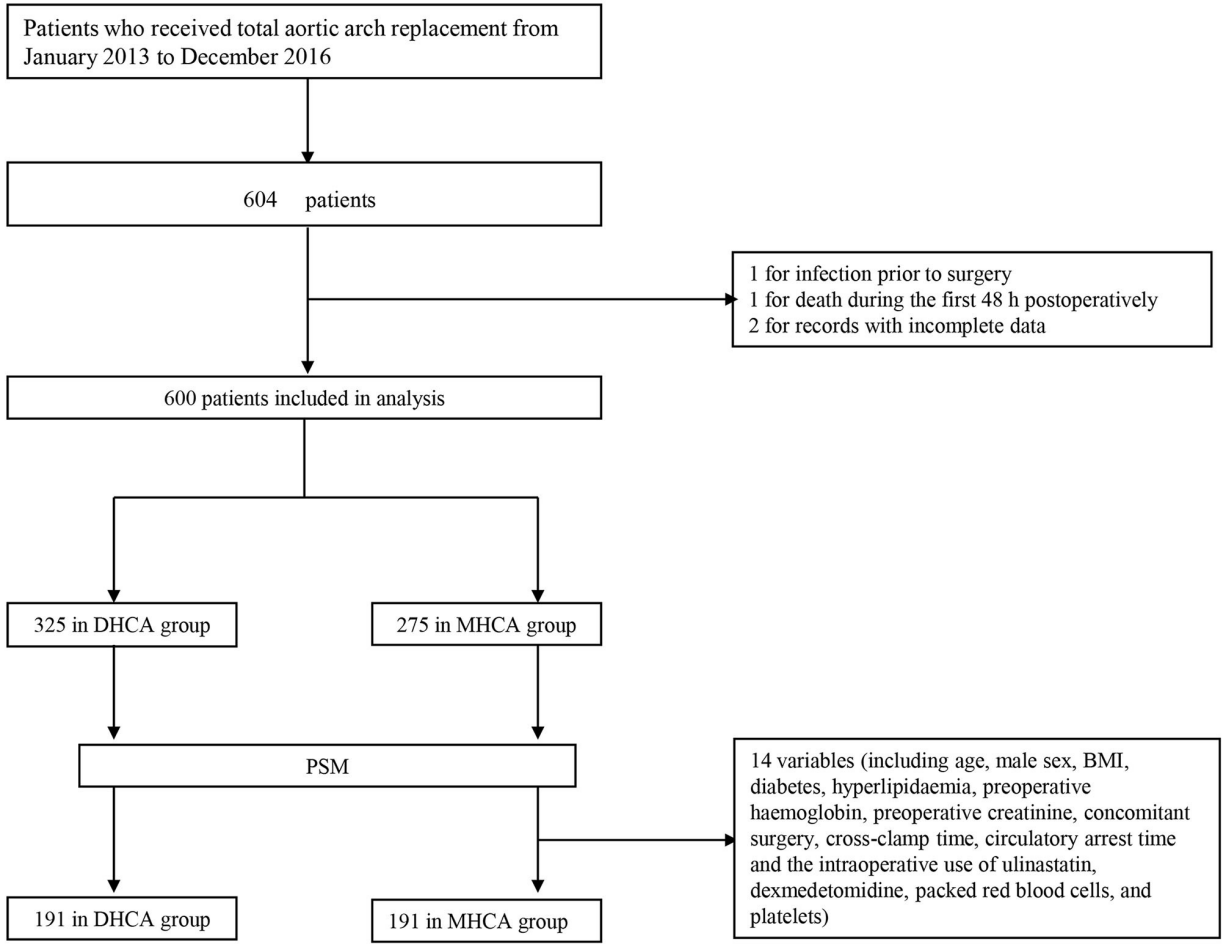
Flowchart. DHCA, deep hypothermic circulatory arrest; MHCA, moderate hypothermic circulatory arrest; PSM, propensity score matching.

A total of 604 consecutive patients who underwent total aortic arch replacement between January 2013 and December 2016 were enrolled. Patients who met any of the following conditions were excluded: (a) infection before surgery, (b) death within 48 h after surgery, and (c) records with incomplete data. Ultimately, four patients were excluded, and data were collected from 600 subjects (Figure 1). Patients were divided into two groups in accordance with the international consensus guideline (9): 14.1–20°C for DHCA and 20.1–28°C for MHCA.

### Clinical Definitions

**Figure 2:**
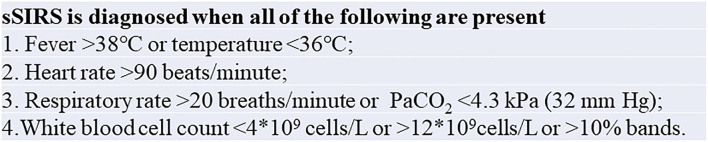
Clinical diagnosis of the sSIRS. sSIRS, severe systemic inflammatory response syndrome.

The primary end point was sSIRS. sSIRS was diagnosed based on the existing American College of Chest Physicians/Society of Critical Care Medicine Consensus Conference if the patient met all four criteria (Figure 2) (10). In our study, sSIRS was observed from 12 to 48 h after admission to the intensive care unit (ICU). We did not evaluate sSIRS in the first 12 h after surgery to avoid false results from systolic drugs, diuretics, and various fluid inputs used immediately after surgery.

The secondary outcomes included in-hospital mortality, use of extracorporeal membrane oxygenation (ECMO), hemofiltration, pulmonary infection, reintubation, tracheotomy, arrhythmia, stroke, paraplegia, gastrointestinal hemorrhage, delirium, rethoracotomy for bleeding, deep sternal wound infection, mechanical ventilation, post-operative length of stay, ICU length of stay, drainage within 24 h, drainage within 72 h, and blood product usage. We identified patients with positive sputum cultures as having pulmonary infections. Stroke was defined as a focal neurological deficit, whether transient or permanent, and confirmed as a new deficit by computed tomography examination. Emergency surgery was defined as surgery performed within 24 h of admission. Prolonged ICU length of stay was defined as a stay in the ICU lasting more than seven days. Hemodialysis was used intraoperatively for every renal failure case. Body mass index (BMI) = weight (kg)/height^2^ (m^2^).

### Operative Methods

Median sternotomy was performed in all patients. The frozen elephant trunk surgical procedure for total arch replacement has been described in detail in previous articles (11). Sun's procedure was used, and the key techniques included implantation of the special open stented graft into the descending aorta, total arch replacement with a four-branched vascular graft, and right axillary artery cannulation (12). All patients were treated with selective antegrade cerebral perfusion for brain protection and early rewarming and reperfusion after distal anastomosis to minimize cerebral and cardiac ischemia. The choice of DHCA or MHCA was at the discretion of the surgeon.

### Intraoperative Management

All patients were administered the anti-inflammatory drug methylprednisolone during surgery for prophylaxis. The additional use of dexmedetomidine or ulinastatin was determined according to the anesthesiologist's assessment of the patient's operative status.

### Statistical Analysis

Statistical analysis was performed using SPSS 26.0 (IBM, Armonk, NY, USA). Categorical data were expressed as counts and percentages. Continuous data were expressed as mean ± SD or as medians and 25–75th percentiles. The likelihood ratio χ^2^-test or Fisher's exact test was used to analyze categorical variables, and Student's *t*-test or Mann-Whitney test was used to analyze continuous variables. Binary logistic regression and multivariable regression analyses were performed with stepwise backward elimination. All statistical analyses were performed at a two-tailed significance level of 0.05. To address missing data, we used a simple attribution method. The missing data were imputed by maximum frequency for categorical variables and by mean or median for continuous variables.

The objective of our study was to compare the incidence of sSIRS between patients who underwent MHCA and those who underwent DHCA, and we expected the other baseline variables to be balanced between the two groups. However, before propensity score matching (PSM), there were significant differences in some baseline variables between the groups, which represented interfering factors. Therefore, we incorporated them into a PSM model to remove their influence. That is, PSM analysis was used to balance covariates. The variables included in the PSM model were those with significant differences between groups at *P* < 0.05 and those significant in the univariate logistic regression analysis at *P* < 0.10. In addition, 14 other variables were selected for inclusion in the PSM model based on practical clinical considerations (including age, male sex, BMI, diabetes, hyperlipidemia, pre-operative hemoglobin, pre-operative creatinine, concomitant surgery, cross-clamp time, circulatory arrest time and the intraoperative use of ulinastatin, dexmedetomidine, packed red blood cells, and platelets). The PSM model was evaluated by multivariate logistic regression to estimate the probability of each patient receiving MHCA treatment. MHCA cases and DHCA control cases were matched by the nearest neighbor algorithm, and the matching criterion (1 to 1 matching) was 0.10. The standardized mean difference (SMD) was used to evaluate the balance of the covariates. After PSM, the characteristics of the patients were compared between the two groups (DHCA and MHCA) using a paired Student's *t*-test or the Wilcoxon signed-rank test for continuous variables and McNemar's test for categorical variables.

## Results

### Demographic Characteristics

A total of 600 patients were included in the final analysis. A total of 275 (45.8%) patients underwent MHCA, and 325 (54.2%) patients underwent DHCA. The incidence of post-operative sSIRS in the no-PSM cohort was 27.3% (164/600). Since some baseline measures differed significantly between the two groups in the no-PSM cohort, we adopted the PSM method to equalize the baseline. After PSM analysis, 191 matched pairs were obtained using the above 14 significant variables (for details of the indicators and rationales, please refer to the Statistical Analysis section). The baseline characteristic PSM-adjusted SMD was smaller (SMD < 0.1) than the unadjusted SMD. Table 1 shows the results of the comparison of demographic data between the two groups.

**Table 1:** Pre-operative characteristics.

**Variables**	**no-PSM**				**PSM**			
	**DHCA**	**MHCA**	**SMD**	***P***	**DHCA**	**MHCA**	**SMD**	***P***
	**(*n* = 325)**	**(*n* = 275)**			**(*n* = 191)**	**(*n* = 191)**		
**Demographic**								
Age (y, mean ± SD)	47.1 ± 10.2	46.4 ± 10.9	0.062	0.433	46.9 ± 9.6	46.1 ± 11.3	0.073	0.483
Male (no.)	240 (73.85%)	215 (78.2%)	0.105	0.216	142 (74.3%)	153 (80.1%)	0.139	0.200
BMI (kg/m^2^, mean ± SD)	25.8 ± 4.3	25.9 ± 4.1	0.026	0.755	25.6 ± 4.1	25.9 ± 4.3	0.069	0.539
**Comorbidity**								
Hypertension (no.)	231 (71.1%)	195 (70.9%)	0.004	0.964	136 (50%)	136 (50%)	0.000	1.000
Diabetes (no.)	4 (1.2%)	15 (5.5%)	0.186	0.003	4 (2.1%)	5 (2.6%)	0.023	1.000
Hyperlipidemia (no.)	71 (21.8%)	83 (30.2%)	0.181	0.020	54 (28.3%)	53 (27.2%)	0.011	1.000
COPD (no.)	4 (1.2%)	9 (3.3%)	0.115	0.087	3 (1.6%)	4 (2.1%)	0.029	1.000
CAD (no.)	16 (4.9%)	12 (4.4%)	0.027	0.746	5 (2.6%)	10 (5.2%)	0.128	0.302
Stroke (no.)	13 (4.0%)	13 (4.7%)	0.034	0.663	9 (4.7%)	6 (3.1%)	0.074	0.581
Smoking (no.)	128 (39.4%)	106 (38.5%)	0.017	0.834	81 (42.4%)	78 (40.9%)	0.032	0.844
Renal failure (no.)	6 (1.8%)	9 (3.3%)	0.080	0.265	5 (2.6%)	4 (2.1%)	0.029	1.000
**Pre-operative cardiovascular status onset**				0.711				0.312
Acute (no.)	254 (78.2%)	222 (80.7%)	0.065		145 (75.9%)	156 (81.7%)	0.146	
Subacute (no.)	37 (11.4%)	29 (10.5%)	0.027		24 (12.6%)	20 (10.5%)	0.068	
Chronic (no.)	34 (10.5%)	24 (8.7%)	0.061		22 (11.5%)	15 (7.9%)	0.130	
Previous cardiovascular surgery (no.)	27 (8.3%)	33 (12%)	0.113	0.133	18 (9.4%)	26 (13.6%)	0.129	0.268
LVEF (%, mean ± SD)	59.6 ± 5.6	59.7 ± 5.6	0.038	0.642	59.9 ± 5.3	59.9 ± 5.3	0.010	0.911
Marfan syndrome (no.)	27 (8.3%)	30 (10.9%)	0.083	0.279	17 (8.9%)	23 (12.0%)	0.101	0.405
**Admission laboratory data**								
Pre-operative Hb (g/L, mean ± SD)	133.5 ± 18.4	134.1 ± 17.0	0.030	0.723	133.5 ± 18.4	134.7 ± 16.8	0.072	0.509
Pre-operative PLT (10^9^/L, mean ± SD)	192.8 ± 71.3	197.2 ± 92.2	0.048	0.512	192.6 ± 75.1	192.4 ± 84.2	0.002	0.987
Pre-operative Glu (mmol/L, mean ± SD)	7.0 ± 1.6	7.2 ± 2.1	0.121	0.099	7.0 ± 1.7	7.1 ± 1.7	0.036	0.668
Pre-operative SCr (mmol/L, mean ± SD)	94.9 ± 35.9	94.2 ± 37.5	0.018	0.825	94.5 ± 36.3	96.2 ± 37.8	0.045	0.660

*Data are presented as the mean ± SD or n (%). BMI, body mass index; CAD, coronary artery disease; COPD, chronic obstructive pulmonary disease; DHCA, deep hypothermic circulatory arrest; Glu, glucose; Hb, hemoglobin; LVEF, left ventricular ejection fraction; MHCA, moderate hypothermic circulatory arrest; PLT, platelet count; PSM, propensity score matching; SCr, serum creatinine; SD, standard deviation; SMD, standardized mean difference*.

### Risk Factors for sSIRS

In the multivariable analysis, cardiopulmonary bypass time (OR 1.007, 95% CI 1.000–1.013; *P* = 0.037) was a predictor for the occurrence of sSIRS following aortic repair, whereas the preferred administration of dexmedetomidine (OR 0.452; 95% CI 0.223–0.914; *P* = 0.027) attenuated it. However, the level of hypothermia (MHCA vs. DHCA) (OR 0.966, 95% CI 0.638–1.464; *P* = 0.872) had no effect on the incidence of sSIRS (Table 2).

**Table 2:** Results of the multivariable analysis of risk factors for sSIRS (*n* = 600).

**Variables**	**OR**	**95% CI**	***P***
CPB time	1.007	1.000–1.013	0.037
Dexmedetomidine	0.452	0.223–0.914	0.027
DHCA vs. MHCA	0.966	0.638–1.464	0.872

*CI, confidence interval; CPB, cardiopulmonary bypass; DHCA, deep hypothermic circulatory arrest; MHCA, moderate hypothermic circulatory arrest; OR, odds ratio; sSIRS, severe systemic inflammatory response syndrome*.

### Comparison of MHCA With DHCA

Patients who underwent DHCA had a significantly higher tendency to undergo concomitant coronary artery bypass grafting (CABG) than patients who underwent MHCA (DHCA 12.3% vs. MHCA 4.4%, *P* = 0.004). To exclude the influence of concomitant CABG on post-operative sSIRS, concomitant surgery was applied as one of the matching indicators. The difference did not persist after PSM. Compared with MHCA, DHCA was associated with a significantly greater cardiopulmonary bypass time (DHCA 188.8 ± 51.4 min vs. MHCA 162.0 ± 44.4 min; *P* < 0.001), operative time (DHCA 392.7 ± 83.9 min vs. MHCA 342.0 ± 85.0 min; *P* < 0.001), aortic cross-clamp time (DHCA 101.3 ± 26.9 min vs. MHCA 93.6 ± 25.5 min; *P* < 0.001) and circulatory arrest time (DHCA 22.6 ± 6.8 min vs. MHCA 19.7 ± 6.2 min; *P* < 0.001). All operative information is shown in Table 3.

**Table 3:** Operative characteristics.

**Variables**	**no-PSM**				**PSM**			
	**DHCA**	**MHCA**	**SMD**	***P***	**DHCA**	**MHCA**	**SMD**	***P***
	**(*n* = 325)**	**(*n* = 275)**			**(*n* = 191)**	**(*n* = 191)**		
Concomitant surgery				0.004				0.696
Ascending aortic repair (no.)	200 (61.5%)	176 (64.0%)	0.051		128 (67.0%)	117 (61.3%)	0.120	
Aortic root operation (no.)	85 (26.2%)	86 (31.3%)	0.110		53 (27.7%)	62 (32.5%)	0.101	
CABG (no.)	40 (12.3%)	12 (4.4%)	0.388		10 (5.2%)	12 (6.3%)	0.051	
Emergency (no.)	210 (64.6%)	157 (57.1%)	0.152	0.060	121 (63.4%)	107 (56.0%)	0.148	0.202
Operation time (min, mean ± SD)	392.7 ± 83.9	342.0 ± 85.0	0.596	<0.001	382.8 ± 86.4	347.1 ± 88.9	0.422	<0.001
CPB time (min, mean ± SD)	188.8 ± 51.4	162.0 ± 44.4	0.603	<0.001	182.3 ± 52.1	168.6 ± 48.2	0.308	0.005
Cross-clamp time (min, mean ± SD)	101.3 ± 26.9	93.6 ± 25.5	0.034	<0.001	98.1 ± 26.8	97.4 ± 27.4	0.027	0.786
Circulatory arrest time (min, mean ± SD)	22.6 ± 6.8	19.7 ± 6.2	0.464	<0.001	21.5 ± 6.8	20.8 ± 6.6	0.104	0.222
Circulatory arrest time≥30 min (no.)	24 (7.4%)	17 (6.2)	0.050	0.561	9 (4.7%)	17 (8.9%)	0.174	0.134
Lowest bladder temperature (°C, mean ± SD)	21.2 ± 2.0	24.9 ± 2.2	1.663	<0.001	21.3 ± 1.9	24.6 ± 2.1	1.451	<0.001
Lowest nasal temperature (°C, mean ± SD)	18.4 ± 1.1	22.3 ± 1.5	2.602	<0.001	18.5 ± 1.0	22.0 ± 1.4	2.345	<0.001
**Intraoperative blood product use**								
PRBC (no.)	196 (60.3%)	146 (53.1%)	0.144	0.075	116 (60.7%)	100 (52.4%)	0.168	0.129
PRBC [u, median (IQR)]	4 (0–6)	2 (0–4)	0.175	0.014	4 (0–6)	2 (0–4)	0.132	0.145
FFP (no.)	141 (43.4%)	116 (42.2%)	0.024	0.767	80 (41.9%)	81 (42.4%)	0.011	1.000
FFP [ml, median (IQR)]	0 (0–600)	0 (0–600)	0.014	0.947	0 (0–600)	0 (0–600)	0.051	0.655
PLT (no.)	302 (92.9%)	250 (90.9%)	0.070	0.365	170 (89.0%)	178 (93.2%)	0.145	0.185
PLT [u, median (IQR)]	2 (1–2)	1 (1–2)	0.430	<0.001	2 (1–2)	2 (1–2)	0.083	0.405
**Intraoperative medications**								
Ulinastatin (no.)	258 (79.4%)	165 (60%)	0.395	0.000	134 (70.2%)	139 (72.8%)	0.053	0.597
Dexmedetomidine (no.)	309 (95.1%)	252 (91.6%)	0.124	0.089	180 (94.2%)	175 (91.6%)	0.094	0.383

*Continuous data are presented as the mean ± SD or median (IQR), and categorical data are presented as n (%). CABG, coronary artery bypass grafting; CPB, cardiopulmonary bypass; DHCA, deep hypothermic circulatory arrest; FFP, fresh frozen plasma; IQR, interquartile range; MHCA, moderate hypothermic circulatory arrest; PLT, platelets; PRBC, packed red blood cells; PSM, propensity score matching; SD, standard deviation; SMD, standardized mean difference*.

Table 4 presents the details of the post-operative outcomes. Post-operative sSIRS was not significantly different between the DHCA and MHCA groups either before or after PSM (no-PSM: *P* = 0.188; PSM: *P* = 0.416), although post-operative sSIRS was more than 4% higher in the DHCA group than in the MHCA group (no-PSM: 29.5 vs. 24.7%; PSM: 29.3 vs. 25.1%) in the no-PSM and PSM cohorts. The rate of gastrointestinal hemorrhage, rate of pulmonary infection and post-operative length of stay were significantly increased in the DHCA group compared with the MHCA group both before and after PSM (*P* < 0.05), but the remaining secondary outcomes were not significantly different between the groups either before or after PSM.

**Table 4:** Post-operative characteristics.

**Variables**	**no-PSM**				**PSM**			
	**DHCA**	**MHCA**	**SMD**	***P***	**DHCA**	**MHCA**	**SMD**	***P***
	**(*n* = 325)**	**(*n* = 275)**			**(*n* = 191)**	**(*n* = 191)**		
**Primary outcome (sSIRS)**	96 (29.5%)	68 (24.7%)	0.111	0.188	56 (29.3%)	48 (25.1%)	0.097	0.416
**Secondary outcomes**								
In-hospital mortality (no.)	5 (1.5%)	7 (2.5%)	0.064	0.380	3 (1.6%)	6 (3.1%)	0.100	0.508
ECMO (no.)	1 (0.3%)	2 (0.7%)	0.049	0.468	0 (0%)	2 (1.0%)	0.123	NA
Hemofiltration (no.)	40 (12.3%)	22 (8.0%)	0.158	0.084	19 (9.9%)	17 (8.9%)	0.039	0.868
Pulmonary infection (no.)	92 (28.3%)	44 (16.0%)	0.335	<0.001	53 (27.7%)	35 (18.3%)	0.257	0.047
Reintubation (no.)	22 (6.8%)	9 (3.3%)	0.196	0.054	14 (7.3%)	8 (4.2%)	0.176	0.286
Tracheotomy (no.)	9 (2.8%)	3 (1.1%)	0.161	0.143	5 (2.6%)	3 (1.6%)	0.101	0.727
Arrhythmia (no.)	41 (12.6%)	22 (8.0%)	0.170	0.066	23 (12.0%)	17 (8.9%)	0.116	0.392
Stroke (no.)	5 (1.5%)	7 (2.5%)	0.064	0.380	3 (1.6%)	6 (3.1%)	0.100	0.508
Paraplegia (no.)	10 (3.1%)	14 (5.1%)	0.091	0.210	4 (2.1%)	12 (6.3%)	0.190	0.077
Gastrointestinal hemorrhage (no.)	13 (4.0%)	3 (1.1%)	0.280	0.028	10 (5.2%)	0 (0%)	0.503	NA
Delirium (no.)	11 (3.4%)	11 (4.0%)	0.031	0.689	6 (3.1%)	8 (4.2%)	0.053	0.791
Rethoracotomy for bleeding (no.)	15 (4.6%)	13 (4.7%)	0.005	0.948	10 (5.2%)	10 (5.2%)	0.000	1.000
Deep sternal wound infection (no.)	7 (2.2%)	1 (0.4%)	0.297	0.057	4 (2.1%)	1 (0.5%)	0.260	0.375
Mechanical ventilation [h, median (IQR)]	23 (15–56)	19 (14–42)	0.353	0.038	21 (14–51)	20 (14–42)	0.348	0.443
Mechanical ventilation >5 d (no.)	31 (9.5%)	14 (5.1%)	0.202	0.039	18 (9.4%)	10 (5.2%)	0.190	0.152
ICU length of stay [d, median (IQR)]	4 (2–6)	2 (2–5)	0.373	0.012	4 (2–6)	3 (2–5)	0.385	0.213
ICU length of stay >7 d (no.)	54 (16.6%)	32 (11.6%)	0.155	0.083	33 (17.3%)	24 (12.6%)	0.147	0.253
Postoperative length of stay [d, median (IQR)]	12 (9–15)	11 (8–13)	0.266	<0.001	13 (10–16)	10 (8–14)	0.339	<0.001
Drainage within 24 h (ml, mean ± SD)	458.2 ± 264.8	505.8 ± 376.4	0.126	0.079	455.0 ± 256.3	502.9 ± 415.4	0.127	0.167
Drainage within 72 h (ml, mean ± SD)	1,219.5 ± 510.0	1,284.9 ± 645.7	0.101	0.175	1,232.6 ± 509.5	1,245.4 ± 616.4	0.142	0.825
**Post-operative blood product use**								
PRBC (no.)	92 (28.3%)	81 (29.5%)	0.025	0.757	57 (29.8%)	52 (27.2%)	0.057	0.665
PRBC [u, median (IQR)]	0 (0–2)	0 (0–2)	0.065	0.736	0 (0–2)	0 (0–2)	0.052	0.815
FFP (no.)	74 (22.8%)	74 (26.9%)	0.093	0.241	47 (24.6%)	56 (29.3%)	0.106	0.349
FFP [ml, median (IQR)]	0 (0–0)	0 (0–200)	0.056	0.250	0 (0–0)	0 (0–400)	0.059	0.394
PLT (no.)	27 (8.3%)	28 (10.2%)	0.062	0.428	16 (8.4%)	20 (10.5%)	0.069	0.597
PLT [u, median (IQR)]	0 (0–0)	0 (0–0)	0.071	0.418	0 (0–0)	0 (0–0)	0.103	0.338
**Laboratory data**								
Post-operative Hb (g/L, mean ± SD)	102.9 ± 14.6	101.8 ± 14.8	0.076	0.353	103.6 ± 14.9	101.5 ± 14.6	0.142	0.160
Post-operative PLT (10^9^/L, mean ± SD)	108.8 ± 51.0	112.2 ± 60.0	0.067	0.383	104.2 ± 47.2	113.9 ± 63.3	0.162	0.100
Post-operative Glu (mmol/L, mean ± SD)	12.1 ± 3.2	12.3 ± 2.9	0.093	0.279	11.9 ± 3.1	12.5 ± 3.0	0.197	0.072
Post-operative SCr (mmol/L, mean ± SD)	198.4 ± 127.3	182.9 ± 121.2	0.128	0.129	187.9 ± 124.2	190.2 ± 125.7	0.019	0.862

*Continuous data are presented as the mean ± SD or median (IQR), and categorical data are presented as n (%). DHCA, deep hypothermic circulatory arrest; ECMO, extracorporeal membrane oxygenation; FFP, fresh frozen plasma; Glu, glucose; Hb, hemoglobin; ICU, intensive care unit; IQR, interquartile range; MHCA, moderate hypothermic circulatory arrest; NA, not available; PLT, platelets; PRBC, packed red blood cells; PSM, propensity score matching; SCr, serum creatinine; SD, standard deviation; SMD, standardized mean difference; sSIRS, severe systemic inflammatory response syndrome*.

## Discussion

This study is the first single-center study with a large sample size to investigate sSIRS after total aortic arch replacement among the type A aortic dissection population. The inflammatory response after cardiac surgery has been widely recognized (13, 14); however, few studies have focused on its occurrence following total aortic arch replacement, especially in sSIRS. Patients with sSIRS have noticeably higher in-hospital mortality than those without sSIRS (15). MacCallum and his colleagues found that 6-month mortality tended to be increased in those with sSIRS (15.5%) relative to those without sSIRS (7.4%) (6). In our study, we found that our in-hospital mortality rate was very low, below 4% in both groups, and did not significantly differ between the DHCA and MHCA groups.

Before PSM, patients with DHCA had lower incidences of diabetes and hyperlipidemia and experienced more concomitant CABG than those in the MHCA group. Moreover, the DHCA group experienced a longer operation time, cardiopulmonary bypass time, cross-clamp time, and circulatory arrest time than the MHCA group. Regarding intraoperative blood transfusion, the DHCA group received more packed red blood cells and platelets than the MHCA group. To generate a suitable control group, PSM was performed. Because aortic cross-clamping time and circulatory arrest time are dependent on the surgical procedure and independent of the temperature of hypothermic circulatory arrest, both factors were included in the matching process. However, operation and cardiopulmonary bypass times were not included in the matching process because those factors are largely dependent on the method of hypothermic circulatory arrest (DHCA vs. MHCA) and the aortic cross-clamping, cooling, and rewarming times. In consideration of the above, 14 variables were selected for inclusion in the PSM model: age, male sex, BMI, diabetes, hyperlipidemia, pre-operative hemoglobin, pre-operative creatinine, concomitant surgery, cross-clamp time, circulatory arrest time and the intraoperative use of ulinastatin, dexmedetomidine, packed red blood cells, and platelets. All of the baseline imbalances disappeared after PSM.

In our study, the overall incidence of sSIRS was 27.3%. Although there was no significant difference in the incidence of sSIRS between the DHCA and MHCA groups, the MHCA group had a slightly (~4%) lower incidence than the DHCA group before and after PSM. Our data suggest that cardiopulmonary bypass time is an independent risk factor for sSIRS, which is consistent with previous studies (16). Moreover, dexmedetomidine may reduce the likelihood of post-operative sSIRS. Such an effect of dexmedetomidine might be possible because this medication is a highly selective α_2_-adrenergic agonist and is commonly used as an anesthetic adjunct for sedation. Related studies have shown that the anti-inflammatory effect of dexmedetomidine may be due to the inhibition of nuclear factor κB activation (17); it might also be related to the slowing effect of dexmedetomidine on heart rate. After PSM, the MHCA group showed low incidences of pulmonary infection and gastrointestinal hemorrhage and a shorter post-operative length of stay than the DHCA group (*P* < 0.05). Furthermore, MHCA reduced the operative time and cardiopulmonary bypass time without increasing mortality or morbidity.

Similar to our study, studies by other groups have shown that among patients undergoing aortic arch surgery, MHCA patients had lower in-hospital and 30-day mortality, shorter cardiopulmonary bypass times, and fewer neurological sequelae than DHCA patients (18). In 2014 (19), the first study to compare MHCA with DHCA in the repair of acute type A dissection was performed and revealed that patients with MHCA have a lower risk of a composite outcome of mortality and major adverse cardiac and cerebrovascular events. In Ma's et al. which included patients who underwent the frozen elephant trunk procedure for type A dissection, MHCA combined with antegrade cerebral perfusion was demonstrated to be a safe and effective method for protecting cerebral and visceral organs (20). In 2020, a study suggested that MHCA can be safely administered to older patients without increasing the risk of serious neurological complications (21). Currently, at Fuwai Hospital, MHCA (nasal temperature, 25°C) plus antegrade cerebral perfusion is used for total aortic arch replacement with the frozen elephant trunk procedure in type A aortic dissection.

Some limitations in our study should be noted. First, this was a single-center retrospective study; therefore, the level of evidence is limited. Second, the identification of sSIRS may be inaccurate because it was based only on the patient's vital signs rather than objective evidence of biochemical test results such as serum C-reactive protein and interleukins. Finally, the study focused only on short-term clinical outcomes; long-term outcomes remain to be explored. Further investigations are encouraged to evaluate the benefits of MHCA during follow-up.

## Conclusions

Our study showed that there was no difference in the development of sSIRS between DHCA and MHCA groups of adult patients with type A aortic dissection who underwent total aortic arch replacement with the frozen elephant trunk procedure at Fuwai Hospital. However, DHCA was related to prolonged cardiopulmonary bypass times and operation times and could increase the rates of pulmonary infection and gastrointestinal hemorrhage as well as the post-operative length of stay. Considering the results of our study and those of other groups, we cautiously recommend the use of MHCA with antegrade cerebral perfusion for most total arch replacements with the frozen elephant trunk procedure in type A aortic dissection.

## Data Availability Statement

The original contributions presented in the study are included in the article/supplementary material, further inquiries can be directed to the corresponding author.

## Ethics Statement

The studies involving human participants were reviewed and approved by the Research Ethics Committees of Fuwai Hospital. Written informed consent for participation was not required for this study in accordance with the national legislation and the institutional requirements.

## Author Contributions

GW designed the study. YD analyzed and interpreted the data and drafted the manuscript. ZF, CZ, and GL assisted in the data processing and analysis. YC, LY, XY, JL, and YS contributed to the data collection. All the authors have read and approved this manuscript.

## Funding

This work was supported by the National Natural Science Foundation of China (Grant No. 81970344).

## Conflict of Interest

The authors declare that the research was conducted in the absence of any commercial or financial relationships that could be construed as a potential conflict of interest.

## Publisher's Note

All claims expressed in this article are solely those of the authors and do not necessarily represent those of their affiliated organizations, or those of the publisher, the editors and the reviewers. Any product that may be evaluated in this article, or claim that may be made by its manufacturer, is not guaranteed or endorsed by the publisher.
