# Corrigendum: Moderate and Deep Hypothermic Circulatory Arrest Have Comparable Effects on Severe Systemic Inflammatory Response Syndrome After Total Aortic Arch Replacement in Patients With Type A Aortic Dissection

**DOI:** 10.3389/fsurg.2022.881715

**Published:** 2022-03-22

**Authors:** Yingjie Du, Zhongrong Fang, Yanhua Sun, Congya Zhang, Guiyu Lei, Yimeng Chen, Lijing Yang, Xiying Yang, Jun Li, Guyan Wang

**Affiliations:** ^1^Department of Anesthesiology, Beijing Tongren Hospital, Capital Medical University, Beijing, China; ^2^Department of Anesthesiology, National Center of Cardiovascular Diseases, Chinese Academy of Medical Sciences and Peking Union Medical College, Fuwai Hospital, Beijing, China; ^3^Department of Anesthesiology, The Affiliated Drum Tower Hospital of Nanjing, University Medical School, Nanjing, China

**Keywords:** severe systemic inflammatory response syndrome, total aortic arch replacement (TAAR), moderate hypothermic circulatory arrest (MHCA), deep hypothermic circulatory arrest (DHCA), type A aortic dissection (TAAD)

The first author was incorrectly spelled as “Yinejie Du.” The correct spelling is “Yingjie Du.”

In the original article, there was an error. In the first sentence of the **Conclusions**, “SIRS should be sSIRS”.

A correction has been made to **Conclusions** section, Paragraph 1:

“Our study showed that there was no difference in the development of sSIRS between DHCA and MHCA groups of adult patients with type A aortic dissection who underwent total aortic arch replacement with the frozen elephant trunk procedure at Fuwai Hospital.”

The authors apologize for this error and state that this does not change the scientific conclusions of the article in any way. The original article has been updated.

## Publisher's Note

All claims expressed in this article are solely those of the authors and do not necessarily represent those of their affiliated organizations, or those of the publisher, the editors and the reviewers. Any product that may be evaluated in this article, or claim that may be made by its manufacturer, is not guaranteed or endorsed by the publisher.

